# A Micromachined Pressure Sensor with Integrated Resonator Operating at Atmospheric Pressure

**DOI:** 10.3390/s131217006

**Published:** 2013-12-10

**Authors:** Sen Ren, Weizheng Yuan, Dayong Qiao, Jinjun Deng, Xiaodong Sun

**Affiliations:** 1 Key Laboratory of Micro/Nano Systems for Aerospace, Ministry of Education, Northwestern Polytechnical University, Xi'an 710072, China; E-Mails: rensen@mail.nwpu.edu.cn (S.R.); dyqiao@nwpu.edu.cn (D.Q.); dengjj@nwpu.edu.cn (J.D.); sxd5275@163.com (X.S.); 2 Shaanxi Province Key Laboratory of Micro and Nano Electro-Mechanical Systems, Northwestern Polytechnical University, Xi'an 710072, China

**Keywords:** pressure sensor, resonant sensor, micromachining, micro resonator, atmospheric pressure, quality factor

## Abstract

A novel resonant pressure sensor with an improved micromechanical double-ended tuning fork resonator packaged in dry air at atmospheric pressure is presented. The resonator is electrostatically driven and capacitively detected, and the sensor is designed to realize a low cost resonant pressure sensor with medium accuracy. Various damping mechanisms in a resonator that is vibrating at atmospheric pressure are analyzed in detail, and a formula is developed to predict the overall quality factor. A trade-off has been reached between the quality factor, stress sensitivity and drive capability of the resonator. Furthermore, differential sense elements and the method of electromechanical amplitude modulation are used for capacitive detection to obtain a large signal-to-noise ratio. The prototype sensor chip is successfully fabricated using a micromachining process based on a commercially available silicon-on-insulator wafer and is hermetically encapsulated in a custom 16-pin Kovar package. Preliminary measurements show that the fundamental frequency of the resonant pressure sensor is approximately 34.55 kHz with a pressure sensitivity of 20.77 Hz/kPa. Over the full scale pressure range of 100–400 kPa and the whole temperature range of −20–60 °C, high quality factors from 1,146 to 1,772 are obtained. The characterization of the prototype sensor reveals the feasibility of a resonant pressure sensor packaged at atmospheric pressure.

## 1. Introduction

Resonant pressure sensors have been widely used in the field of precision pressure measurement in recent years for their advantages in accuracy and long-term stability. Furthermore, resonant pressure sensors allow a direct interface with digital circuits and are suitable for remote transmission because of their quasi-digital output signals. Resonant pressure sensors have several significant advantages over other conventional micro-electro-mechanical-systems (MEMS) pressure sensors [1,2]. The accuracy and stability of piezoresistive pressure sensors and capacitive pressure sensors, for example, are limited by the sensors' electronics and interface circuits. These sensors are highly susceptible to electrical noise and electrical drift, which result in inaccuracy and degradation of long-term stability. By contrast, the long-term drift of resonant pressure sensors primarily arises from the mechanical property changes of the resonators. It is well accepted that the accuracy and stability of well-designed resonant pressure sensors are an order of magnitude better than those of piezoresistive pressure sensors and capacitive pressure sensors [3].

As the heart of a typical resonant pressure sensor, a resonator is used as a resonant strain gauge to detect the deflection of the pressure-sensitive diaphragm. To obtain a high mechanical quality factor (Q-factor) for high performance, the resonator is normally encapsulated in a vacuum to decrease the gas damping effects. A variety of technologies have been exploited for the vacuum encapsulation of resonant pressure sensors. Glass tube evacuation technology was used in the RPT Series products made by GE Druck [4–6]. Silicon direct fusion bonding technology was used in the 8000 Series products made by GE Druck [3] and in the P90 sensors made by Thales Avionics [7]. A unique process that was specially developed by Yokogawa Electric employed selective epitaxial growth, selective anisotropic etching and hydrogen evacuation technology [8,9]. Anodic bonding [10], traditional weld sealed vacuum metal packaging [11–15] and adhesive vacuum bonding [16] were also used. Moreover, there are still other vacuum packaged resonant pressure sensors [17–20], including designs that use getter materials [21]. Although these sensors achieve high performance levels, often with 0.01% full scale (FS) overall measurement accuracy for a high Q-factor on the order of 10^4^ [3,5,8], the vacuum packaging of resonant pressure sensors is very complex and fairly costly.

To reduce the packaging complexity and cost, it is widely accepted that other resonant sensors, such as Coriolis vibratory rate gyroscopes and resonant accelerometers, are usually packaged at atmospheric pressure. Bosch's Z-axis gyroscopes [22] with Q-factors of 1,200, Analog Devices' iMEMS ADXRS series gyroscopes [23] with Q-factors of 45 and SA30 resonant accelerometers of SensoNor [24] with Q-factors greater than 1,000 are classic cases of resonant sensors that are successfully packaged at atmospheric pressure. A resonant pressure sensor that allows the resonator to maintain direct contact over a measured pressure media range of 0–120 kPa has also been reported [25,26]. At atmospheric pressure, only a low Q-factor of the resonator can be achieved, but a low Q-factor does not mean low accuracy. The accuracy of a resonant sensor is also affected by the signal-to-noise ratio (SNR) of the sensor chip and the feedback control circuit. Traditional vibrating-wire densimeters with Q-factors of less than 100 but much greater signal amplitudes can even attain an accuracy of 0.05% [27,28]. Furthermore, a resonant pressure sensor packaged at atmospheric pressure will still have good long-term stability, similar to a sensor packaged in a vacuum [29] owing to the inherent characteristic that its mechanical properties dominate the long-term drift.

This paper proposes an electrostatically comb-driven resonant pressure sensor that is designed to hermetically package the micromechanical resonator in dry air at atmospheric pressure. The sensor is designed to realize low-cost resonant pressure measurement with medium accuracy. The design, fabrication process and initial characterization results of a prototype sensor are presented. Experimental results demonstrate the feasibility of the new resonant pressure sensor design, and a high Q-factor is obtained.

## 2. Sensor Design

**Figure 1.**
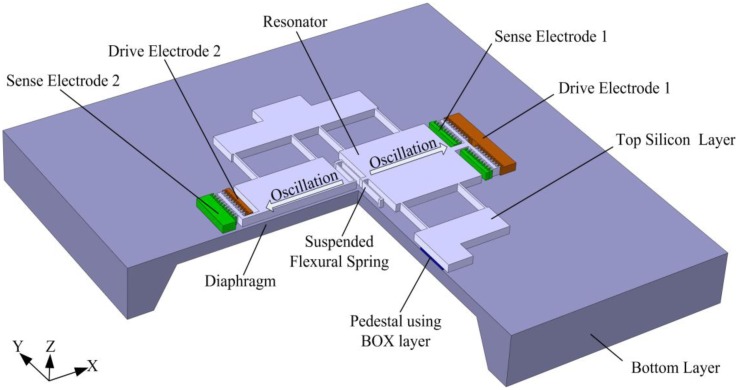
Schematic drawing of the resonant pressure sensor.

The structure is based on a commercially available silicon-on-insulator (SOI) wafer because of its high yield and simple yet reliable fabrication process. In addition, the monocrystalline top silicon layer allows a very high intrinsic Q-factor of the resonator material. The resonator is suspended above the center of the diaphragm with two pedestals fixed on the diaphragm's surface. The diaphragm is made out of the bottom layer, and the pedestals are formed using the buried oxide (BOX) layer. To minimize the gas-damping effects between the resonator and the diaphragm, a BOX layer of 4 μm is selected, which is the thickest layer available in standard commercial SOI wafers. Moreover, electrostatic excitation and capacitive detection are used. When the pressure of the measured media increases, the deflection of the pressure-sensitive diaphragm alters the resonator's stiffness, which results in an increase of its resonant frequency. Figure 1 shows the schematic drawing of the resonant pressure sensor. The resonator is an improved double-ended tuning fork (DETF) structure with two inertial movable plates supported by eight stress-sensitive beams and a suspended flexural spring.

As the resonator is encapsulated at atmospheric pressure, the Q-factor of the resonant pressure sensor is limited. To optimize the performance of the resonant pressure sensor, two main efforts have been made. First, the Q-factor should be as high as possible. A high Q-factor plays a crucial role not only because it improves the sensitivity and resolution of the resonant pressure sensor but also because it simplifies the feedback control circuit and decreases the influence of unwanted external interference. To eliminate the perturbing effect of the feedback control circuit, a Q-factor greater than 1,000 is needed [30]. Second, the SNR of the sensor chip should also be as large as possible, which can effectively enhance the accuracy and resolution of the resonant pressure sensor. A large SNR means to have a small noise disturbance and a simplified interface circuit. Accordingly, the total error from the sensor chip and the circuit is decreased.

There are two main methods for obtaining a large SNR: signal amplitude increment and noise reduction. To increase the signal amplitude of the sensor chip, a thick top silicon layer of 60 μm is chosen as the resonator layer, and electrostatic combs are microfabricated using high aspect ratio deep reactive ion etching (DRIE). To reduce noise disturbance, differential electrostatic combs are used as the drive and sense elements, and an electromechanical amplitude modulation (EAM) method is used for capacitive detection.

### 2.1. Quality Factor of the Resonator

The Q-factor of the resonator is defined as the ratio of the total energy stored in the resonator to the sum of energy loss per cycle:

(1) $$Q=2\pi \frac{W}{\Delta W}=\frac{m\omega }{c}=\frac{k}{c\omega }$$

where *W* is the total energy stored in the resonator, Δ*W* is the sum of energy loss per cycle, *k* is the spring constant of the resonator, *ω* is the radial frequency of the resonator, *c* is the coefficient of damping force, m is the effective mass of the resonator and given by *m* = *m_p_* + *m_f_* + 13*m_b_*/35, and *m_p_*, *m_f_*, and *m_b_* are the total mass of the movable plates, the total mass of the movable fingers, and the total mass of the stress-sensitive beams and the suspended flexural spring, respectively.

To improve the Q-factor, the total stored energy should be increased and the energy loss per cycle should be reduced. For the designed resonant pressure sensor, the effective mass and spring constant of the resonator should be increased and the damping coefficient should be reduced, as the resonant frequency is predetermined to be 35 kHz.

An additional suspended flexural spring is introduced into the resonator structure to increase the effective mass and the spring constant as indicated in Figure 1. The spring attaches the two inertial movable plates together at a central position. Thus, the two parts of the resonator are mechanically coupled as a whole, which ensures that there is only a single working vibration mode and a single resonant frequency, along with the presence of slight imbalances in the electrostatically driven force and the microfabricated structure of the resonator. Furthermore, the first two vibration modes of the resonator will be separated further apart. It is beneficial to reduce the mechanical coupling between the desired dynamically balanced out-of-phase mode (mode 2) and the first in-phase mode (mode 1). Nevertheless, the spring constant of the suspended flexural spring should be limited, considering the fact that the suspended flexural spring can decrease the stress sensitivity of the resonator.

Reducing the energy dissipation from damping is the chief method used to raise the Q-factor. There are basically four major damping mechanisms acting on the resonator by which energy is lost, and each damping mechanism can be relevant to a specific Q-factor. The overall Q-factor is given by:

(2) $$\frac{1}{Q}=\frac{1}{Q_{a}}+\frac{1}{Q_{c}}+\frac{1}{Q_{i}}+\frac{1}{Q_{s}}$$

where *Q_a_*, *Q_c_*, *Q_i_* and *Q_s_* are associated with the viscous damping from the surrounding gas, the mechanical coupling damping of the resonator, the internal damping due to the resonator's material properties, and the surface damping effects, respectively.

**Figure 2.**
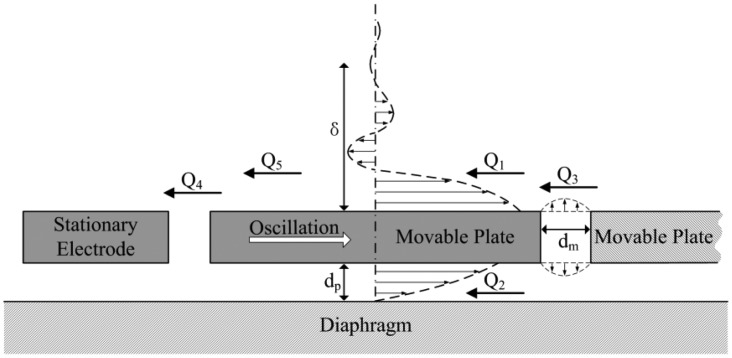
The gas damping distribution in half of the DETF resonator.

The gas viscous damping associated with *Q_a_* is the dominant energy loss mechanism, as the resonator is designed to be packaged at atmospheric pressure. It is affected by the nature and pressure of the encapsulated gas, the geometries and vibration modes of the resonator, and the proximity of the adjacent surfaces. Gas viscous damping is normally classified into three categories: slide-film gas damping, squeeze-film gas damping and gas drag damping. Because the resonator is symmetrical and oscillates in a dynamically balanced out-of-phase mode, only half of the DETF resonator is characterized analytically. Figure 2 shows the gas damping distribution in half of the resonator, with the dashed line representing the fluid velocity profile. There are five main damping force components, and each one can be related to a specific Q-factor as well. *Q_1_* and *Q_2_* arise from the slide-film gas damping above and underneath the movable plate; *Q_3_* arises from the squeeze-film gas damping among the movable plates, the stationary electrodes and the suspended flexural spring; *Q_4_* arises from the slide-film gas damping on the sidewalls of the movable fingers and the squeeze-film gas damping between the tips and bases of the fingers; and *Q_5_* arises from the gas drag damping.

The penetration depth *δ* is an important characteristic of the oscillating gas, and the slide-film gas can be further modeled as Couette flow or Stokes flow by comparing *δ* with the typical dimension *d*. The penetration depth is defined as the distance where the amplitude of gas velocity decays to 1% of that at the movable plate surface and can be approximated as [31]:

(3) $$\delta =\sqrt{\frac{2\mu }{\rho_{\text{air}}\omega }}$$

where *μ* is the viscosity coefficient of air and *ρ_air_* is the density of air.

The penetration depth is a function of the resonant frequency and is approximately 11 μm for the designed resonant pressure sensor. Regarding the slide-film gas damping above the movable plate, because *d* is much greater than *δ*, the gas can be modeled as Stokes flow, and *Q_1_* can be expressed as [31,32]:

(4) $$Q_{1}=\frac{m\omega \delta }{\mu A}$$

where *A* is the effective top area of the resonator, given by *A* = *A_p_* + *A_f_* + 0.5*A_b_*, and *A_p_*, *A_f_* and *A_b_* are the top area of the movable plate, the top area of the movable fingers, and the top area of the stress-sensitive beams and the suspended flexural spring, respectively.

For the slide-film gas damping underneath the movable plate, the distance *d_p_* is 4 μm, which is much smaller than *δ*. Therefore, the gas undergoes Couette flow and *Q_2_* is derived by [31,32]:

(5) $$Q_{2}=\frac{m\omega d_{p}}{\mu A}$$

The squeeze-film gas damping can be described by the combined effect of the viscous damping force related to the viscous flow and the elastic damping force related to the gas compression. Both of these forces are dependent on the resonant frequency. Because the resonant frequency of the designed resonator is well below the cut-off frequency, the elastic damping force is negligible and the coefficient of squeeze-film gas damping associated with the viscous damping force can be written as [33]:

(6) $$c=\frac{64\sigma P_{a}lh}{\pi^{6}\omega d_{m}}\sum_{\underset{\text{odd}}{m,n}}\frac{m^{2}+{\left(n/\beta \right)}^{2}}{{\left(mn\right)}^{2}\left\{{\left[m^{2}+{\left(n/\beta \right)}^{2}\right]}^{2}+\sigma^{2}/\pi^{4}\right\}}$$

where *P_a_* is the static ambient pressure, *l* is the length of squeeze-film surface, *h* is the thickness of the resonator, *d_m_* is the static gap between squeeze-film surfaces, *β* = *l*/*h* is the aspect ratio of squeeze-film surfaces, $\sigma =\frac{12\mu h^{2}\omega }{P_{a}{d_{m}}^{2}}$ is the squeeze number, and m and n are odd integers.

Consequently, *Q_3_* can be computed, as *d_m_* is predetermined to be 40 μm to restrain the notching effect in DRIE [34] and reduce squeeze-film gas damping:

(7) $$Q_{3}=\frac{m\omega }{c_{3}}=\frac{m\omega }{c_{ss}+c_{ps}+c_{pe}}$$

where *c_ss_*, *c_ps_*, and *c_pe_* represent the squeeze-film damping coefficients within the suspended flexural spring, between the movable plates and the suspended flexural spring, and between the movable plates and the stationary electrodes, respectively.

Considering the comb fingers, the gas damping consists of the slide-film gas damping on the sidewalls of the movable fingers and the squeeze-film gas damping between the tips and bases of the fingers, so *Q_4_* can be calculated as:

(8) $$\frac{1}{Q_{4}}=\frac{1}{Q_{4\text{sidewall}}}+\frac{1}{Q_{4\text{tip}}}=\frac{\mu A_{\text{overlap}}}{m\omega g}+\frac{c_{tb}}{m\omega }$$

where *g* is the comb finger gap, *A_overlap_* is the total area of the comb finger overlaps, and *c_tb_* is the squeeze-film damping coefficient between the tips and the bases of the movable fingers and the stationary fingers.

There is also gas drag force damping acting on the movable plates because a velocity gradient exists from the boundary layer to the more distant points in the surrounding gas. *Q_5_* can be approximated as [31]:

(9) $$Q_{5}=\frac{m\omega }{10.7\mu l_{\text{drag}}}$$

where *l_drag_* is the characteristic dimension of the movable plates, which is one half of the movable plate width.

The overall Q-factor resulting from the viscous damping of the surrounding gas can be estimated by combining Equations (4), (5), (7–9). The predicted Q-factor should be multiplied by a factor of approximately 0.5 [35], as the edge effect and finite-size effect of the resonator are ignored:

(10) $$\frac{1}{Q_{\text{estimate}}}=\sum_{i=1}^{5}\frac{1}{Q_{i}}=\frac{\mu }{m\omega }(\frac{A}{\delta }+\frac{A}{d_{p}}+\frac{A_{\text{overlap}}}{g}+\frac{c_{ss}+c_{ps}+c_{pe}+c_{tb}}{\mu }+10.7l_{\text{drag}})$$

(11) $$Q_{a}\approx \frac{1}{2}Q_{\text{estimate}}$$

The coupling damping associated with *Q_c_* is a type of structural damping and can be reduced by proper sensor design. There are two sources of vibration energy loss. One source is the internal mode coupling between the working mode and the adjacent modes, and the other source is the external mechanical coupling between the pedestals and diaphragm of the resonant pressure sensor and the environment. To minimize the internal mode coupling, the working mode frequency should be far away from the adjacent modes and the Q-factor of the working mode should be as large as possible. At the same time, the Q-factor of the adjacent modes should be as small as possible. According to our experience, a frequency separation greater than 10% of the working mode frequency is enough for a Q-factor of 1,000. To minimize the external mechanical coupling, a lateral resonator vibrating in a dynamically balanced out-of-phase mode is chosen and the resonator is mounted at the nodes of the chosen vibration mode. Due to symmetrical structure and symmetrical vibration, the center of gravity is unchanged and the counterforces and the bending moments nearly balance out at the nodes, which can significantly alleviate the vibration energy losses at the fixed positions. In addition, the mechanical coupling between the resonator and the diaphragm is further reduced as their working modes are perpendicular, which means the sensor is less susceptible to surface contaminations and the stability of the sensor is enhanced. For a well-designed, dynamically-balanced, out-of-phase lateral resonator working at atmospheric pressure, the coupling damping is negligible.

The internal damping associated with *Q_i_* relies on the dislocations, purity, microfabrication defects and thermo-elastic damping (TED) of the material used. As the top silicon layer of the SOI wafer is an ideal high Q-factor monocrystalline material, the dislocations and the impurity are negligible. In addition, the microfabrication defects can be neglected as well using annealing. As a result, the Q-factor due to TED (*Q_TED_*) dominates *Q_i_*, which is given as [36]:

(12) $$Q_{\text{TED}}=(\frac{f^{2}+{f_{T}}^{2}}{f\;f_{T}})\frac{C_{p}\;\rho }{\alpha^{2}TE}$$

(13) $$f_{T}=\frac{\pi \lambda }{2C_{p}\;\rho_{\text{silicon}}w^{2}}$$

where *f* is the resonant frequency of the resonator, *f_T_* is defined as the thermo-elastic frequency, *w* is the width of the vibrating beam, *T* is the absolute temperature in the equilibrium state, *C_p_* is the heat capacity at constant pressure, and *α*, *E*, *λ*, and *ρ_silicon_* are the thermal expansion coefficient, elasticity modulus, thermal conductivity, and density of silicon, respectively.

According to the above equation, *Q_TED_* is determined only by the vibrating beam width and resonant frequency of the resonator. Considering the nonlinear behavior of vibration and the separation of vibration modes, the stress-sensitive beam is 15 μm wide and the suspended flexural spring is 8 μm wide. Consequently, *Q_TED_* can be calculated as approximately 9 × 10^4^, which implies a very high *Q_i_*, and the internal damping can be ignored.

The surface damping effects associated with *Q_s_* are mostly caused by surface defects and surface contaminations. Surface damping generally dominates in resonators with submicron-scale features. The dimensions of the resonator in this study are relatively large, on the order of hundreds of micrometers, and the resonator is designed to vibrate in dry air. So the surface-to-volume ratio is small and there are little absorbates on the surface. As a result, the surface energy dissipation can be omitted in the resonator. As a consequence, the entire Q-factor of the resonator can be estimated from Equations (2), (10) and (11):

(14) $$\frac{1}{Q}=\frac{2\mu }{m\omega }(\frac{A}{\delta }+\frac{A}{d_{p}}+\frac{A_{\text{overlap}}}{g}+\frac{c_{ss}+c_{ps}+c_{pe}+c_{tb}}{\mu }+10.7l_{\text{drag}})$$

**Figure 3.**
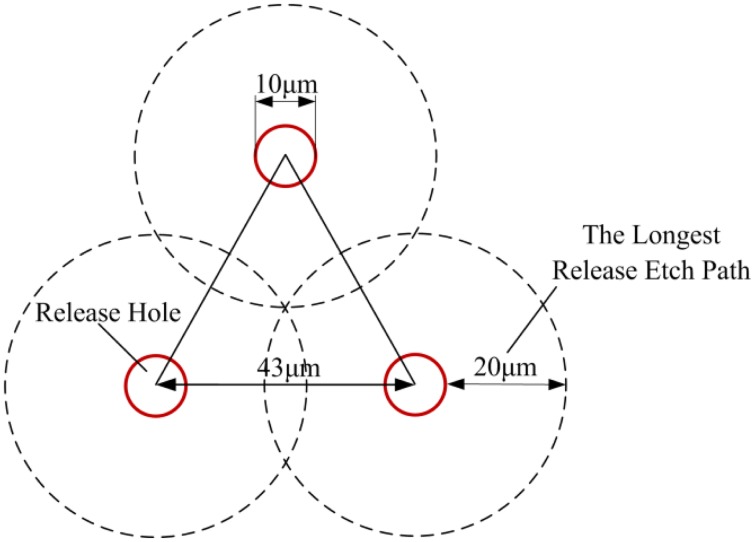
Schematic structure of the release hole arrangement.

According to Equation (14), the ratio between the effective mass *m* and the effective top area *A* of the movable plates should be maximized to obtain a high Q-factor. Considering the structure is based on an SOI wafer, carefully arranged release holes through the top silicon layer are needed to release the large movable plates of the resonator uniformly and preserve the fixed pedestals intact. However, there is almost no lateral flow of gas in the release holes and the trapped gas oscillates together with the movable plates. Therefore, the total area of the release holes is actually added to the effective top area of the movable plates and should be minimized [31]. Based on our experience, the longest release etch path must not be more than 20 μm for circular release holes with 10 μm diameters. Consequently, an equilateral triangle arrangement is selected as shown in Figure 3. The distance between the centers of the release holes is 43 μm and the ratio of the effective areas between the release holes and the movable plates is reduced to 4.87%.

### 2.2. Sensor Structure

Because the stress-sensitive beams and the suspended flexural spring vibrate together in the desired dynamically balanced out-of-phase mode (mode 2) and the nonlinear vibration behavior is ignored for relatively small vibration amplitude, the resonant frequency at the working mode of the resonator can be expressed as:

(15) $$f=\frac{1}{2\pi }\sqrt{\frac{\frac{8Eh{w_{b}}^{3}}{{l_{b}}^{3}}+\frac{4Eh{w_{\text{s}}}^{3}}{{l_{\text{s}}}^{3}}+\frac{48Nhw_{b}}{5l_{b}}}{m_{p}+m_{f}+\frac{13}{35}m_{b}}}=f_{0}\sqrt{1+\frac{12Nw_{b}{l_{b}}^{2}{l_{\text{s}}}^{3}}{5E(2{l_{\text{s}}}^{3}{w_{b}}^{3}+{l_{b}}^{3}{w_{\text{s}}}^{3})}}$$

where *l_b_* and *w_b_* represent the length and width of the stress-sensitive beam, *l_s_* and *w_s_* represent the length and width of the suspended flexural spring, and *N* is the stress in the axial direction of the stress-sensitive beam caused by the bending of the diaphragm.

The vibration amplitude of the resonator at the resonant frequency is:

(16) $$\left|X\right|=Q\frac{F}{k}$$

where *F* is the drive force of the electrostatic combs.

To measure the drive capability of the resonator, Equation (16) is converted to:

(17) $$\frac{\left|X\right|}{F}=\frac{Q}{k}=\frac{1}{\omega c}$$

**Figure 4.**
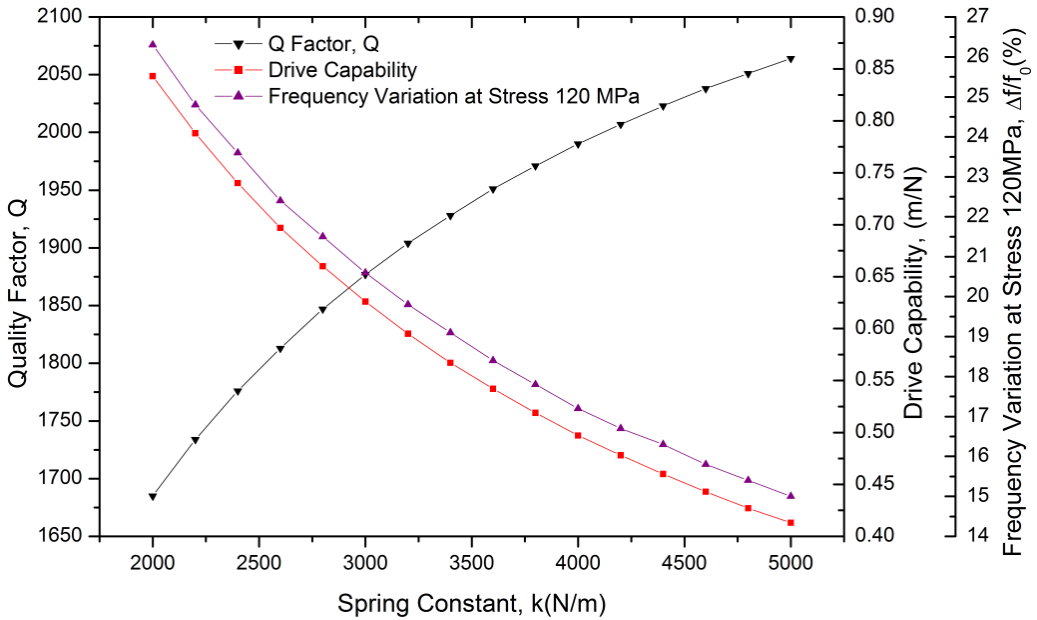
The relationships between the Q-factor, frequency variation at full scale stress of 120 MPa, drive capability and spring constant of the resonator.

There are 50 fingers on each of the two drive combs and the two sense combs, with gap, width, length, and overlap dimensions of 3, 3, 30 and 10 μm, respectively. As a result, the physical dimensions of the resonator can be derived at different spring constants. Moreover, the Q-factor, stress sensitivity and drive capability of the resonator can be calculated as well using Equations (14), (15) and (17). The relationships between the Q-factor, frequency shift at full scale stress of 120 MPa, drive capability and spring constant of the resonator are plotted in Figure 4.

According to Figure 4, the Q-factor of the resonator increases as the spring constant increases, while the drive capability and frequency shift of the resonator decrease. A trade-off has been made and a resonator spring constant of 3,600 N/m has been chosen. The predicted frequency shift at the full scale stress of 120 MPa is 18.40% and the Q-factor is expected to be approximately 1,951. With a DC bias of 10 V and AC excitation of 2 V_rms_, a vibration amplitude of 0.19 μm can be estimated.

Finite element analysis was performed using ANSYS. A simplified three-dimensional finite element model of the resonant pressure sensor, including the resonator, the diaphragm and the connecting pedestals, has been created to simulate the sensor characteristics. Then, the geometries of the resonator were further optimized, with the dimensions of the pressure-sensitive diaphragm set to 3,100 μm × 3,100 μm × 65 μm. The primary design parameters of the resonant pressure sensor are listed in Table 1.

**Figure 5.**
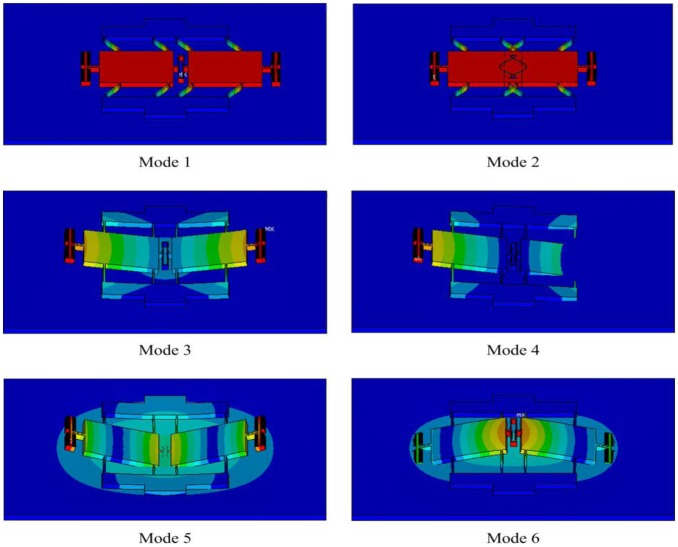
The first six vibration mode shapes of the resonant pressure sensor simulated using ANSYS.

**Figure 6.**
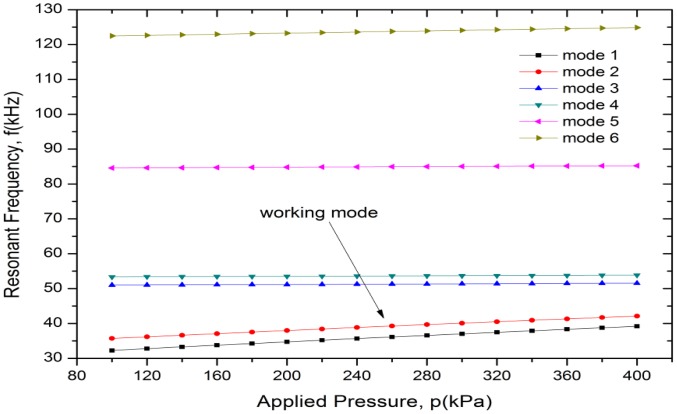
Resonant frequency variations of the first six vibration modes versus applied pressure.

Figure 5 gives the first six vibration mode shapes of the resonant pressure sensor, and the resonant frequency variations of the first six vibration modes with applied pressure are depicted in Figure 6; higher-order vibration modes are not of concern. The resonator oscillates in a dynamically balanced out-of-phase mode and is parallel to the diaphragm, which is in accordance with the Q-factor designed above. Additionally, the working mode is well separated from the adjacent modes over the full scale pressure range of 100∼400 kPa.

## 3. Fabrication

**Figure 7.**
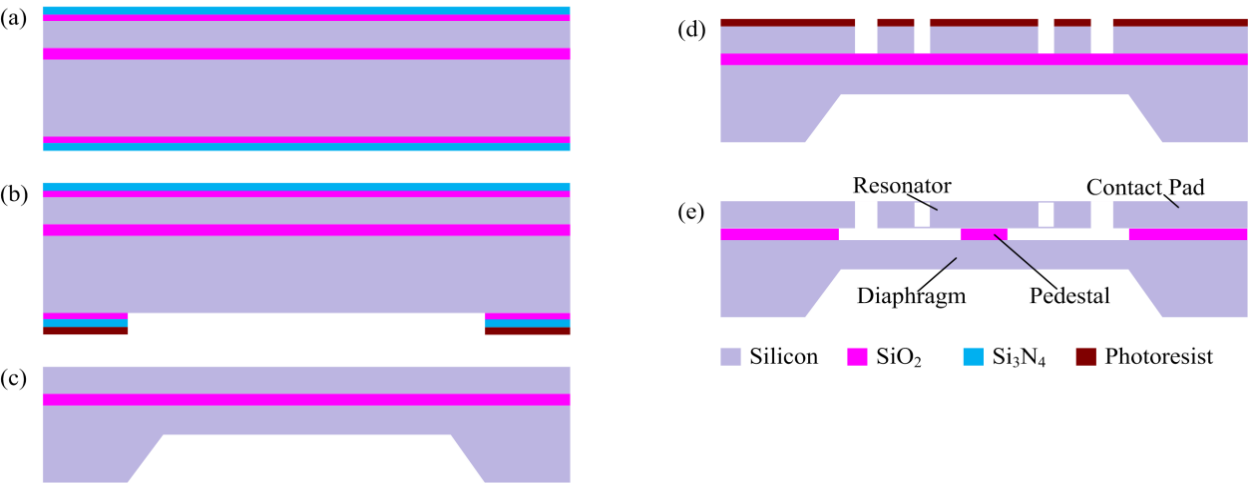
Fabrication process of the resonant pressure sensor.

**Figure 8.**
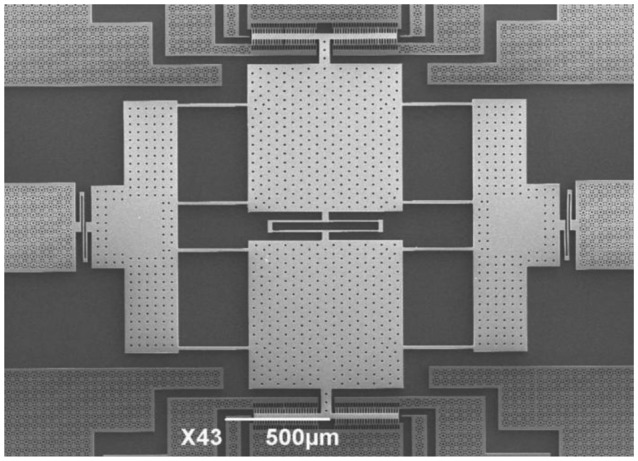
SEM picture showing the completely released resonator structure.

The sensor is fabricated using a <100> p-type SOI wafer. The top silicon layer (60 μm thick, 0.01∼0.02 Ω cm) is used as the resonator layer; the BOX layer (4 μm thick) is used as the sacrificial layer and the pedestal layer; and the bottom layer (400 μm thick) is used as the diaphragm and frame layer. A dimension compensation of 0.5 μm has been made to eliminate the undercut effect of DRIE. Details of the resonant pressure sensor fabrication process are shown in Figure 7a–e. First, a 440 nm oxide is grown on both sides of the wafer by thermal oxidation, followed by a 200 nm low pressure chemical vapor deposition (LPCVD) using Si_3_N_4_ (see Figure 7a). Then, the anisotropic etching mask is defined on the backside of the wafer using SF_6_ plasma etching (see Figure 7b). Next, a timed anisotropic etching in a tetramethylammonium hydroxide (TMAH) solution is applied and the diaphragm is formed, followed by the removal of the oxide and Si_3_N_4_ with HF solution (see Figure 7c). After that, using Shipley S1818 positive photoresist as the etching mask, DRIE is performed to etch through the top silicon layer from which the resonator is formed (see Figure 7d). Finally, the BOX layer underneath the resonator is selectively removed using an HF solution for the final device release (see Figure 7e). The sensor chip has lateral dimensions of approximately 5.5 mm × 5.5 mm. Figure 8 shows the scanning electron microscope (SEM) picture of the completely released resonator structure.

**Figure 9.**
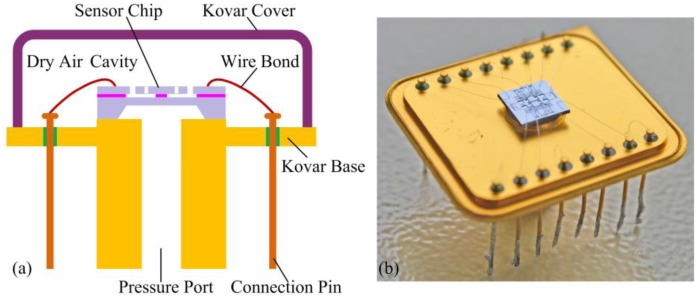
(**a**) Schematic drawing of the resonant pressure sensor package. (**b**) Picture of the resonant pressure sensor package before hermetic sealing.

Figure 9a is the schematic of the resonant pressure sensor package design. The sensor chip is mounted onto a custom 16-pin Kovar base with an epoxy resin, which lets the diaphragm just cover the pressure port while being exposed to the measured pressure media (see Figure 9b). After wire bonding, each sensor is tested using a Polytec MSA-500 micro system analyzer for initial selection. Then, the Kovar cover is hermetically sealed in dry air at atmospheric pressure by resistance welding. As a result, the resonator is isolated from moisture and particle contamination.

## 4. Characterization Results

**Figure 10.**
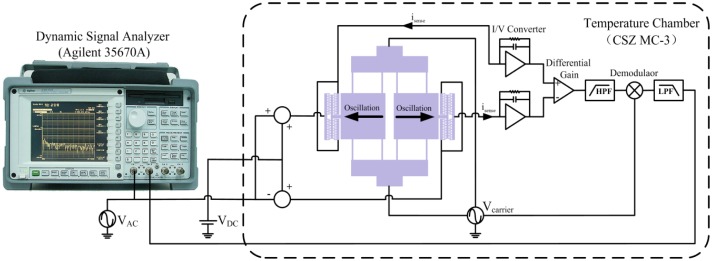
Schematic diagram of the electrical measurement system.

The open loop frequency response characteristics of the resonant pressure sensor are tested by an electrical measurement system based on the Agilent 35670A dynamic signal analyzer, as illustrated in Figure 10. A Mensor CPC8000 precision pneumatic pressure controller is used to generate standard pressure with a high precision within ±0.01% of reading, while a CSZ MC-3 environmental test chamber is used to supply the temperature environment with a regulating accuracy of ±0.5 °C. As has been mentioned earlier, differential sense elements and EAM are used for capacitive detection to obtain a large SNR. The high frequency carrier signal of 3.5 V_rms_ at 1.25 MHz is applied to the two inertial movable plates and is generated by a Tektronix AFG3252 arbitrary function generator. In addition, an average of ten continuous test results has been made to improve the measurement precision.

**Figure 11.**
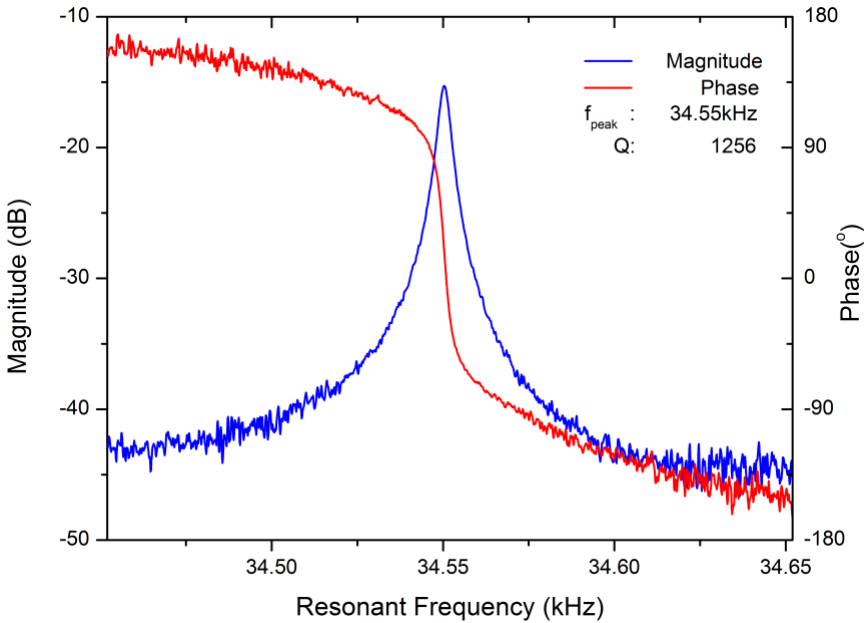
Resonance characteristics of the resonant pressure sensor at atmospheric pressure.

Preliminary measurements show that the resonant pressure sensor has a fundamental frequency of approximately 34.55 kHz at 20 °C and 100 kPa, which agrees well with the simulation. However, the Q-factor is typically 1,256, which is much lower than the predicted value. The resonance characteristics of the resonant pressure sensor at atmospheric pressure are shown in Figure 11. We believe this is mainly caused by the fact that the edge effect and the finite-size effect in the micromechanical resonator are drastically underestimated, especially in the squeeze-film gas damping. The substrate proximity effect and the closed regions between the squeeze-film surfaces created by the resonator geometries make it very difficult for the trapped gas to flow, which results in a much bigger viscous damping force and a much higher energy dissipation. In addition, there are other influences which have corresponding negative impacts on the Q-factor, such as the notching effect at the bottom edges, sidewall microfabrication defects resulting from DRIE, small unbalanced oscillations due to the inevitable asymmetry of the electrostatically driven force and the microfabricated resonator structure, and a gas temperature rise owing to the heat produced by mechanical vibration.

**Figure 12.**
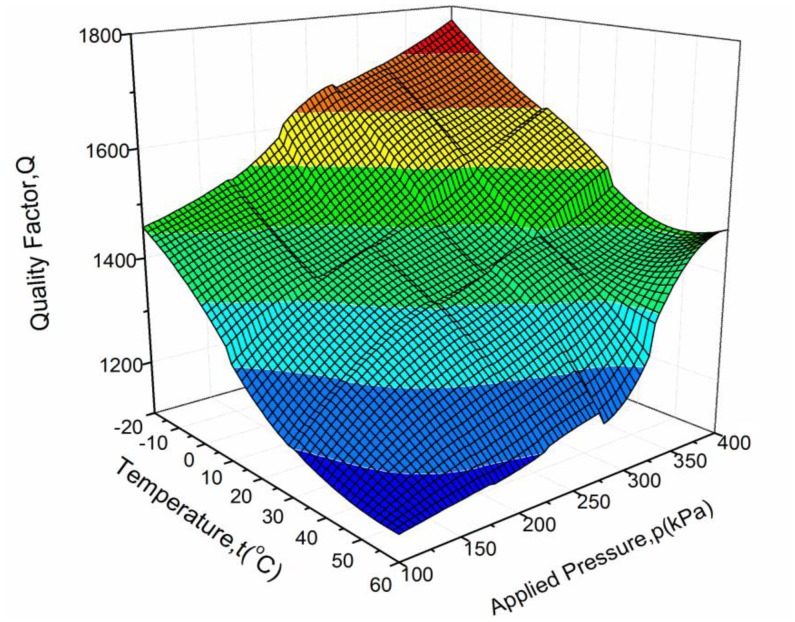
The variation of Q-factor with applied pressure and ambient temperature.

The Q-factors of the resonator over the full scale pressure of 100∼400 kPa and the whole temperature range of −20∼60 °C are further tested, with results ranging from 1,146 to 1,772 as shown in Figure 12.

It can be observed that the Q-factor increases with increasing applied pressure and decreasing temperature. When the applied pressure increases, the resonant frequency of the resonator goes up, while the distance between the resonator and the diaphragm reduces slightly. The total stored energy increases greatly relative to the incremental energy loss, which causes the increase in the Q-factor. With rising temperature, the viscosity coefficient of air increases greatly. In addition, the resonant frequency decreases slightly because the resonant pressure sensor has a negative temperature coefficient. As a result, the Q-factor decreases. The Q-factor characteristics with applied pressure and ambient temperature are consistent with Equation (14).

**Figure 13.**
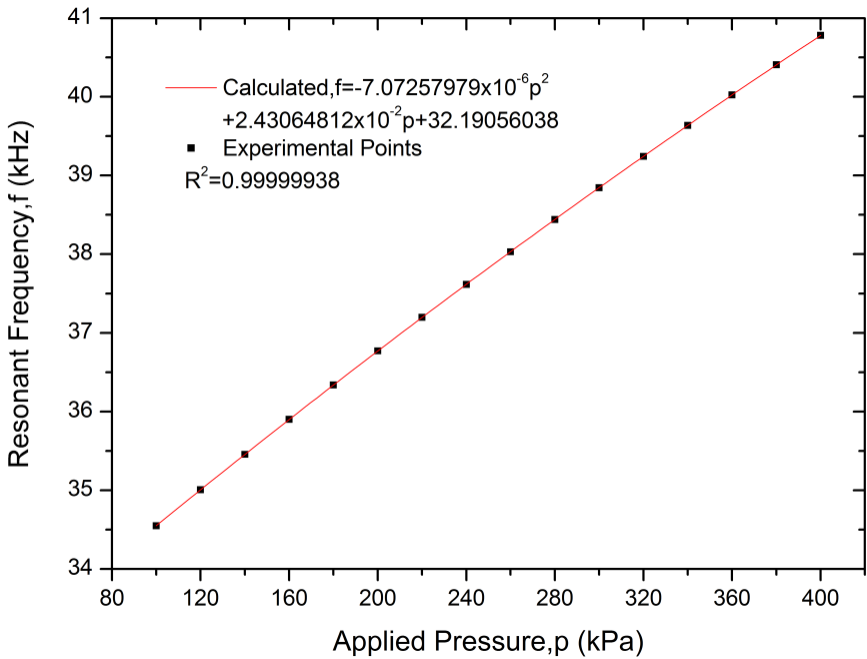
Resonant frequency with applied pressure at 20 °C.

The variation of resonant frequency with applied pressure is shown in Figure 13 along with a second-order polynomial curve fit that has an extremely high correlation coefficient. Measurements show that the resonant pressure sensor has a pressure sensitivity of approximately 20.77 Hz/kPa and changes by 18.04% at the full scale pressure, with nonlinearity of 0.045% FS, a hysteresis error of 0.14% FS, and a repeatability error of 0.18% FS. The pressure sensitivity agrees well with the predicted value, but the hysteresis error and repeatability error are not satisfactory. That is because the epoxy resin used in the sensor package deforms when the diaphragm deflects with applied pressure. This deformation transmits stress between the sensor chip and the Kovar base, which introduce serious hysteresis error and repeatability error into the resonant pressure sensor and deteriorates the stability of the sensor.

**Figure 14.**
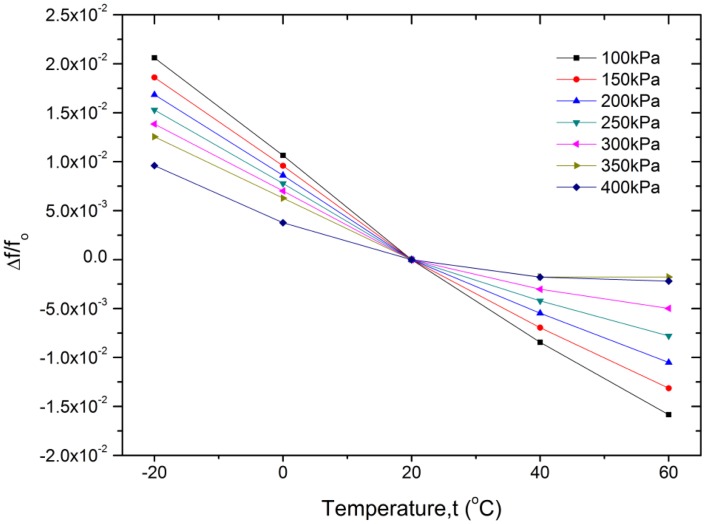
Temperature characteristics of the resonant pressure sensor at different applied pressures.

Figure 14 shows the temperature characteristics of the prototype sensor. Over the temperature range of −20∼60 °C, the average temperature sensitivity of the resonator is between −0.015%/°C (at 400 kPa, and relative to the initial frequency at 20 °C) and −0.046%/°C (at 100 kPa, and relative to the initial frequency at 20°C), which means the temperature coefficient of the resonant pressure sensor varies from −0.082%/°C to −0.25%/°C when considering the frequency change at full scale pressure. The thermal shift is much larger than that of traditional resonant pressure sensors [18,37]. The primary reason is that the reference pressure of the hermetically packaged air is more susceptible to temperature change as its volume is almost constant, which can lead to a significant shift of the resonant frequency. Additionally, there is a thermal expansion coefficient mismatch between the sensor chip, the epoxy resin and the Kovar base, which will result in stress across the resonator when the temperature changes. The operating temperature range is restricted by the material properties of the epoxy resin. The temperature coefficient is also a function of the applied pressure, as the geometrical shape and stress distribution of the resonant pressure sensor change with different pressure loads. Further studies are currently underway to improve the performance of the resonant pressure sensor. The hysteresis error, repeatability error and temperature coefficient of the resonant pressure sensor can be reduced significantly by well stress isolation [38,39]. Furthermore, temperature compensation can be made using an incorporated temperature sensor [5,40]. Accordingly, the medium accuracy of approximately 0.05% FS is expected.

## 5. Conclusions

A resonant pressure sensor based upon an improved micromechanical DETF resonator has been presented, which uses electrostatic comb excitation and capacitive detection. The performance of the prototype sensor demonstrates the feasibility of a medium accuracy resonant pressure sensor with a micromechanical resonator vibrating at atmospheric pressure. A Q-factor greater than 1,100 has been accomplished over the full scale pressure range of 100–400 kPa and the whole temperature range of −20–60 °C. The main advantages of this type of resonant pressure sensor are the elimination of vacuum encapsulation and the simple yet reliable fabrication process based on a commercially available SOI wafer. These advantages significantly reduce the packaging complexity and cost. However, there is a large negative temperature coefficient in the resonant pressure sensor. An incorporated temperature sensor should be used for temperature compensation, as the hermetically packaged air is more susceptible to temperature change. Further studies are currently underway to improve the performance of the resonant pressure sensor.

## Figures and Tables

**Table 1.** Primary design parameters of the resonant pressure sensor.

**Parameters**	**Value**
Pedestal thickness	4 μm
Diaphragm dimensions	3,100 μm × 3,100 μm × 65 μm
Resonator thickness	60 μm
Stress-sensitive beam width	15 μm
Stress-sensitive beam length	325 μm
Suspended flexural spring width	8 μm
Suspended flexural spring length	231 μm
Finger gap	3 μm
Finger width	3 μm
Finger length	30 μm
Finger overlap	10 μm
Finger number	50 × 4
